# Dimensional effect of graphene nanostructures on cytoskeleton‐coupled anti‐tumor metastasis

**DOI:** 10.1002/SMMD.20230014

**Published:** 2023-08-03

**Authors:** Qiqige Du, Na Li, Jiaqi Lian, Jun Guo, Yi Zhang, Feng Zhang

**Affiliations:** ^1^ Wenzhou Institute University of Chinese Academy of Sciences Wenzhou China; ^2^ Key Laboratory of Optical Technology and Instrument for Medicine Ministry of Education University of Shanghai for Science and Technology Shanghai China; ^3^ Shanghai Advanced Research Institute Chinese Academy of Sciences Shanghai China

**Keywords:** actin cytoskeleton, cancer cell migration, dimensional effects, graphene‐based nanomaterials, real‐time polymerization

## Abstract

Interactions between inorganic materials and living systems can be strongly influenced by the dimensional property of the materials, which can in turn impact biological activities. Although the role of biomaterials at the molecular and cellular scales has been studied, research investigating the effects of biomaterials across multiple dimensional scales is relatively scarce. Herein, comparing the effectiveness of two‐dimensional graphene oxide nanosheets (GOs) and three‐dimensional graphene oxide quantum dots (GOQDs) (though not zero‐dimensional because of their significant surface area) in cancer therapies, we have discovered that GOs, with the same mass concentration, exhibit stronger anti‐cancer and anti‐tumor metastasis properties than GOQDs. Our research, which employed liquid‐phase atomic force microscopy, revealed that lower‐dimensional GOs create a more extensive nano‐bio interface that impedes actin protein polymerization into the cytoskeleton, leading to the prevention of tumor metastasis. These results help to better understand the underlying mechanisms and offer a dimensional perspective on the potential of optimizing the properties of graphene‐based materials for clinical applications, e.g., cancer therapy.

## Graphical abstract

A dimensional perspective on the potential of optimizing the properties of graphene‐based materials for clinical applications was proposed. Lower‐dimensional graphene oxide nanosheets (GOs) with the same mass concentration exhibit stronger anti‐cancer/tumor metastasis properties than higher‐dimensional graphene oxide quantum dots in that GO creates a more extensive bio‐nano interface that impedes actin protein polymerization into cytoskeleton, leading to the prevention of tumor metastasis.

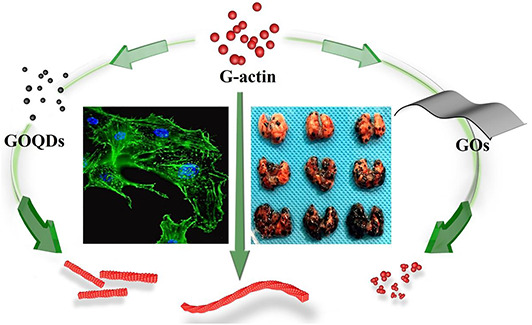

**Key points**

- Real‐time atomic force microscope revealed the process of actin cytoskeleton formation on the nanoscale.
- Dimension of graphene‐based nanomaterials significantly influences cytoskeleton‐coupled anti‐tumor metastasis.
- Two‐dimensional graphene oxide nanosheets showed stronger inhibition efficiency than three‐dimensional globular graphene oxide quantum dots.

## 1 INTRODUCTION

The use of nanomaterials in cancer therapy has become increasingly popular due to their highly effective ability to inhibit cancer cell migration through the unique nano‐bio interface formed between nanomaterials and cancer cells. By designing and manipulating this interface, we can not only gain a better understanding of the interaction between nanomaterials and cancer cells but also create new opportunities for the development of anti‐cancer materials. The cytoskeleton, a complex network of protein fibers that provides structural support and aids in cell movement and division, is a heavily researched target for anticancer therapies, as changes to it can promote tumor growth and metastasis. Actin, a cytoskeletal protein that exists in two forms, globular actin (GA) and its linearly polymeric counterpart, F‐actin (FA), is of particular interest.^1^, ^2^ The polymerization of monomeric GA into polymeric FA is a crucial process that has significant implications for various physiological processes, such as cell division,^3^ differentiation,^4^ and migration.^5^ However, this process can also contribute to harmful processes, such as tumor metastasis,^6^, ^7^ which is responsible for the majority of cancer‐related deaths. Therefore, inhibiting tumor metastasis is considered as a promising approach to cancer therapy.^8^, ^9^, ^10^

Numerous efforts have been made to develop anti‐cancer nanomaterials, and graphene oxide nanosheets (GOs) have been found to disrupt the polymerization of GA through various mechanisms. These include disrupting native contacts of two actin subunits,^11^ isolating actin trimers,^12^ increasing cytokine and chemokine production,^13^ and decreasing cell adhesion.^14^ Simulation results have also shown that functional groups on GOs can form hydrogen bonds with oxygen‐containing residues in actin, leading to actin disassembly.^15^ Although reports have demonstrated that graphene‐based nanomaterials (GBNs) can interfere with cancer cell migration by disrupting the polymerization of GA,^12^, ^16^, ^17^ further research is needed to fully understand the potential of these materials for cancer therapy.^18^ The relationship between GBNs and the cytoskeleton in the context of cancer metastasis is complex and still not well understood. Further research is needed to elucidate the underlying mechanisms and assess the potential risks and benefits of using GBNs in cancer with their physicochemical properties, e.g., structural dimension, which could also play key roles in coupling with biological systems ranging from molecules to cells and higher levels; however, the corresponding research is relatively scarce.

To investigate how the structural dimensions of biomaterials affect biological systems, we selected two typical GBNs: two‐dimensional GOs, and three‐dimensional graphene oxide quantum dots (GOQDs). The reason why we termed GOQDs as three‐dimensional materials but not zero‐dimensional is due to their significant surface area that can interact with biomolecules. We systematically studied the dimensional dependency of the anticancer and antitumor effects of these two kinds of GBNs. Our results showed that GBNs with lower dimensions exhibited more potent antitumor and anticancer effects than those with higher dimensions due to the expansion of the nano‐bio interface for coupling biomolecules with GBNs. We focused on how GBNs of different dimensions affect actin polymerization‐related tumor metastasis and elucidated the in‐depth mechanism of how GBNs interfere with the cytoskeleton and prevent tumor metastasis through in vitro and in vivo experiments, which were across molecular conformation and cellular morphology analyses. This study provides unique insights into the dimensional bioeffects of GBNs, which could inspire the rational design of nanomaterials for clinical applications.

## 2 RESULTS AND DISCUSSIONS

### 2.1 Real‐time observing how GBN dimension influences actin polymerization

**FIGURE 1:**
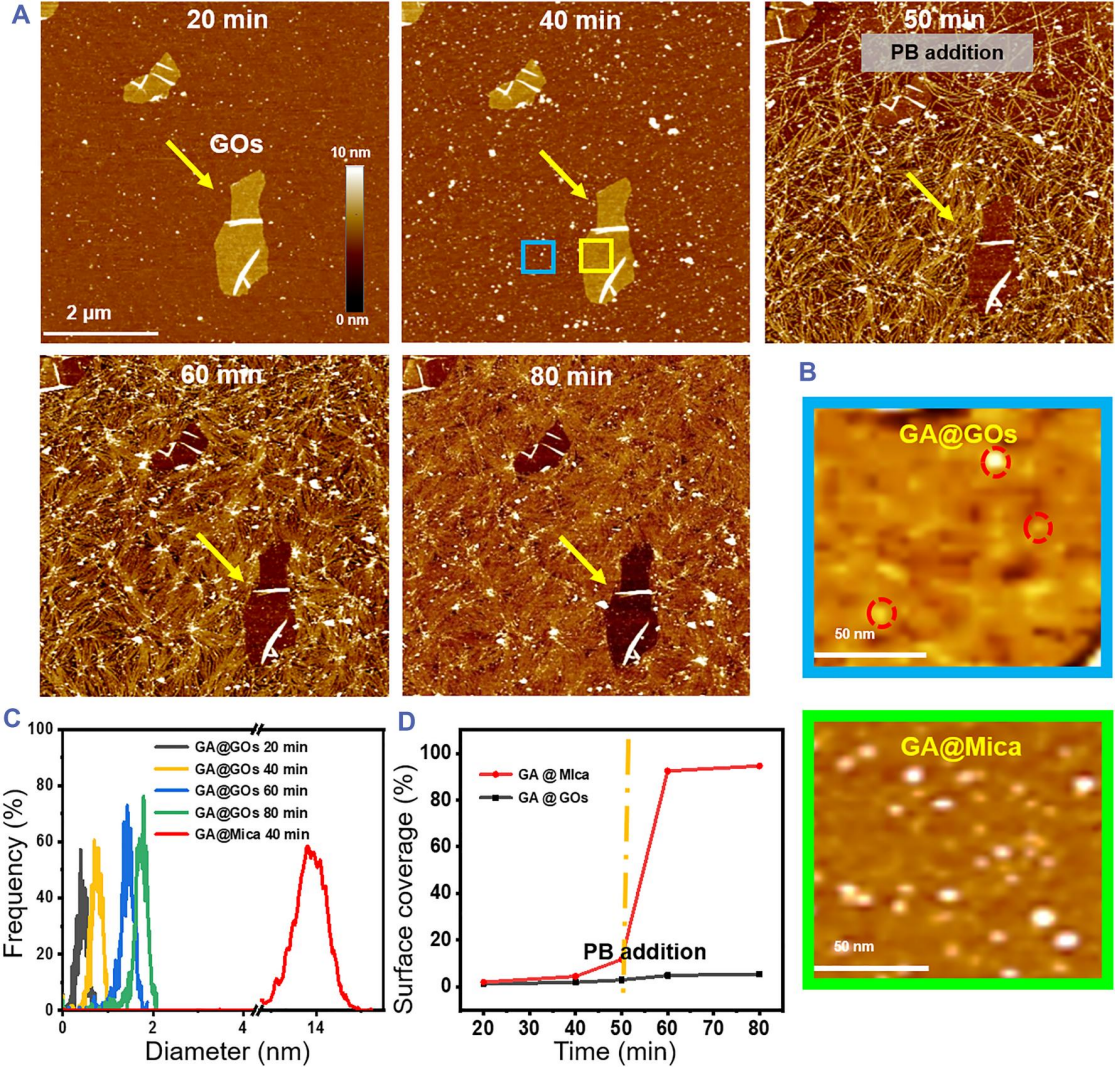
Real‐time observation of actin polymerization on two‐dimensional substrates. (A) Snapshots of in situ liquid phase AFM observation of GA polymerization on the mica‐GOs hybrid surface. GA (40 μM) and PB were added in at 10 and 50 min, respectively. (B) The magnified images of blue and green boxed region in 40 min. (C) Dynamic particle size distribution of GA aggregates on mica (data the blue boxed region) and GOs (data the green boxed region) GA. (D) Surface coverage rate plots of mica and GOs in the green and blue boxed region from 0 to 80 min, respectively. AFM, atomic force microscope; GA, globular actin; GOs, graphene oxide nanosheets; PB, polymerization buffer.

The in situ liquid‐phase atomic force microscope (AFM) was employed to trace the polymerization process of actin proteins. As depicted Figure 1A and Figure S1, we began imaging the mica and GOs (labeled by yellow arrow) grafted substrate in the general buffer to achieve a stable imaging condition, then GA was steadily added in a titration‐like manner.^19^ With increasing incubation time, monomeric and oligomeric GA may occupy the surface of mica and GOs in different ways. Interestingly, GA adsorbed on mica without any apparent variation, whereas the GA captured by GOs were significantly different (labeled by the red circles in Figure 1B).^20^, ^21^ We speculated that GOs have the capacity to remodulate the structure of GA. After 40 min incubation, the diameter of GA on mica gradually increased up to 14 nm; in contrast, the GA adhered on the GOs instead presented a much smaller diameter below 1 nm (Figure 1C), suggesting the collapsed nature of the GA adhered on the GOs. Also, such collapse observed here appeared to be well consistent with the remodulated creased β‐sheet content confirmed by the circular dichroism (CD) analysis,^22^ and synergistic effects could make such collapse more significant.^23^ Once the polymerization of GA was initiated by the addition of polymerization buffer (PB) as an initiator, GA rapidly polymerized into FA on the mica surface (Figure 1A). However, all the GA absorbed on the surface of GOs only exhibited a very limited size increase from 0.4 nm for 20 min incubation to 1.8 nm for 80 min incubation and no appreciable polymerization events were observed (Figure 1A,C). The adsorption kinetic curve shown in Figure 1D further confirmed that all the GA adsorbed on mica could respond significantly to PB. However, the GA adsorbed on GOs was inert to the polymerization initiator.

The dynamic process of GA polymerization in the presence of GOQDs was also recorded (Figure S2). In the presence of GOQDs and PB, GA could only polymerize some short filaments after 30 min incubation. These freakish filaments were short and scattered, and they could be depolymerized in a short time, which indicated that these filaments is weaker than native filament bundles. The different assembled structures of GA explored by AFM showed the effect of dimension‐dependent regulation by GOQDs/GOs on the polymerization of GA.

### 2.2 Reveal the dimension dependent coupling between actin and GBNs

**FIGURE 2:**
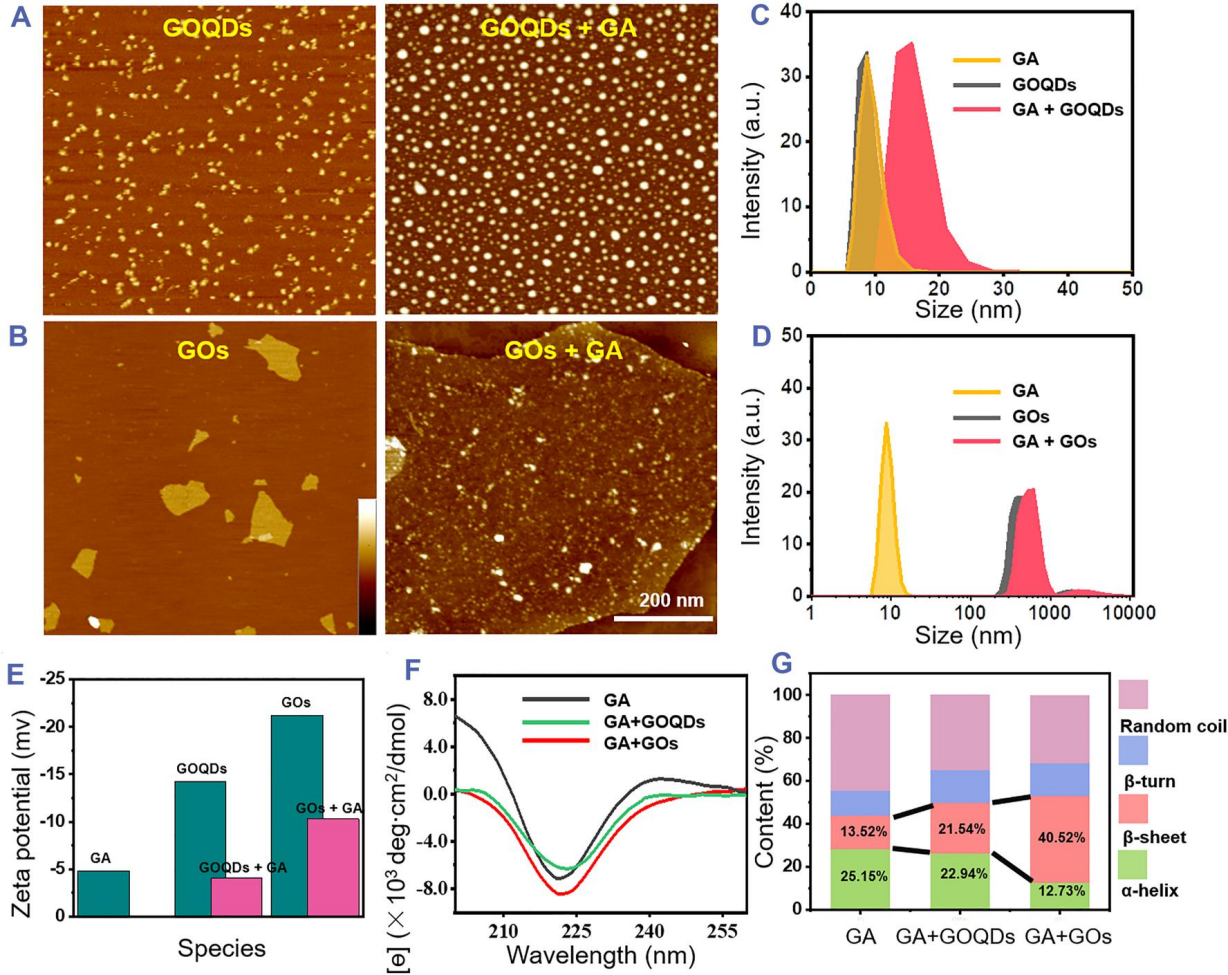
Dependency of material dimension on the nano‐bio coupling. AFM images of (A) GOQDs before and after mixed with GA, and (B) GOs before and after mixed with GA. The hydrodynamic size distributions of GA before and after incubation with (C) GOQDs and (D) GOs. (E) The zeta potential comparison of GA, GOQDs, GOs and their mixtures. (F) Before and after incubation with GOQDs and GOs, CD spectroscopy was performed on GA. (G) The quantity analysis of secondary structure contents from (F). The 1 microscale bar fits for the left of both (A and B), the 200 nm scale bar fits for the right of (A and B); and the height bar inset in (B) fit for all AFM images with a range of 1–10 nm. AFM, atomic force microscope; CD, circular dichroism; GA, globular actin; GOQDs, graphene oxide quantum dots; GOs, graphene oxide nanosheets.

Considering the pivotal role of dimensional properties in various physiological processes, here two representative graphene nanomaterials including GOQDs and GOs were specified to establish the different dimensional bioeffects between nanomaterials and GA and thus dissected the correlation between lower/higher dimensions and GA polymerization. First, the used GOQDs and GOs were characterized by dynamic light scattering (DLS) and AFM. Here, GOQDs are present as dot‐like geometry and have a narrowly distributed size around 9 nm (Figure 2A,C). The GOs have a sheet‐like shape and a relatively broad size distribution ranging from 300 to 600 nm (Figure 2B,D). Consequently, it is possible to speculate that the dot‐like GOQDs and the sheet‐like GOs are two excellent GBNs for establishing a different dimensional interaction with GA.

DLS was first employed to reveal the size change before and after the mixture of GA with these two nanomaterials. As shown in Figure 2C, GOQDs shared a similar size of ∼8 nm with GA; upon mixing the GOQDs with GA, the increased size up to 17 nm of the mixture suggested the formation of nano‐bio interface between GOQDs and GA. Similarly, an appreciable increase in the size accompanied by the mixture of GOs with GA also implied the establishment of nano‐bio interface between GOs and GA (Figure 2D).^24^ In addition, the zeta potential assay showed that both the GOQDs and GOs were demonstrated to be negatively charged species before GA was added (Figure 2E). The decrease in the zeta potential caused by the addition of GA suggested the formation of a complex between GA and two nanomaterials, with the more appreciable zeta potential decrease involved in the mixture of GA with GOs. The above‐mentioned results indicate that the binding of GA with GBNs is the primary inhibitory mechanism from GA to FA, and the GOs demonstrate stronger inhibitory than GOQDs, which is due to the contrasting nano‐bio interface of them.

Next, CD spectroscopy was employed to evaluate the effect of the dimensional coupling effect on the conformation of GA by monitoring its secondary structure changes (Figure 2F). The CD analysis of pure GA predominantly showed as α‐helix. Upon the addition of GOQDs or GOs, an appreciable decrease in absorption intensity emerged, suggesting that the α‐helix content of GA decreased, which is often associated with the unfolding of GA backbones, with the addition of GOs causing a more apparent decrease. The small‐sized GOQDs exhibit higher curvature, and the nano‐bio interface area (exposed) will be greatly reduced compared with GOs. Hence, GOQDs showed less decrease in α‐helix content of GA than GOs. Further, the analysis outcome on the secondary structure content showed that (Figure 2G) after co‐incubating the GA with GOQDs/GOs, the α‐helix content declined from 25.1% for pure GA to 22.94% for GA mixed with GOQDs and 12.37% for GA mixed with GOs. In turn, the β‐sheet content increased from 13.52% for pure GA to 21.54% for GA mixed with GOQDs and to 40.52% for GA mixed with GOs. As is known, the secondary structure of the protein is stabilized mainly by H‐bonding, hydrophobic and other non‐covalent interactions. As a result of the aforementioned secondary structure changes, the remodulated structure of GA could be obtained by adding GOQDs and GOs, with the established nano‐bio interface disrupting the inherent non‐covalent interactions involved in GA protein. The sufficient nano‐bio interfaces of GOs are equipped to show a more striking transformation in the GA structure. GA remodulated with GOs showed apparent changes in secondary content, such as an appreciable decrease in α‐helix content and a pronounced increase in β‐sheet, all these changes might result from the strong hydrophobic interactions between the aromatic amino acid residues in GA and the extended conjugated surface of GOs together with H‐bonding interaction between amino acid residues of GA and hydroxyl groups present on GOs.^25^ Notably, such strong interaction between GA and GOs will trigger a significant unfolding of GA, which will in turn enhance their interactions due to the enlarged nano‐bio interface, leading to the tight absorbance of GA on the surface of GOs and finally significantly impede the polymerization of GA. However, the small‐sized GOQDs were found unable to significantly alter the secondary structure content of GA due to its failure to establish a sufficient nano‐bio interface with GA. As a result, the GOQDs could not impede the polymerization of GA when compared with GOs.

**FIGURE 3:**
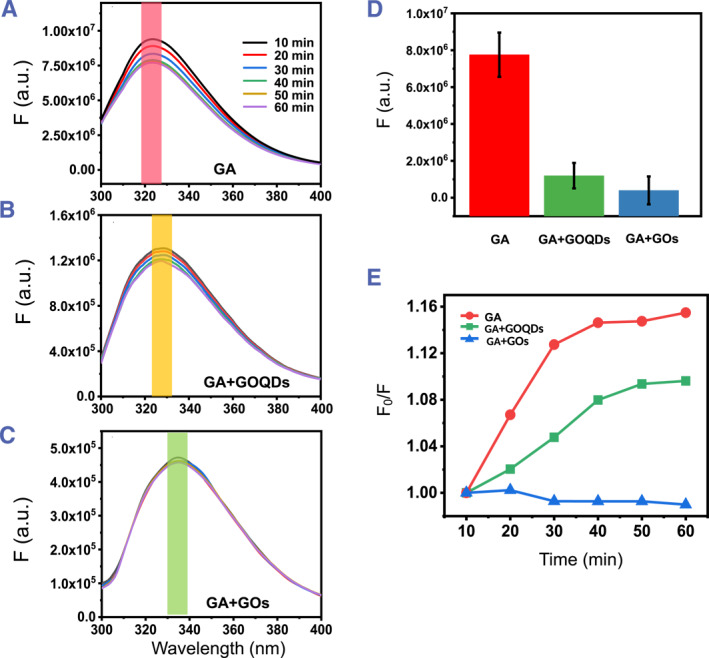
Fluorescence spectroscopy for coupling actin with GO‐material. Dynamic fluorescence spectra of (A) GA, GA mixed with (B) GOQDs and (C) GOs. (D) Comparison of the average peak values (marked with a colorful ribbon) of (A–C) in 60 min. (E) Stern–Volmer plots for the quenching efficiencies in (A–C). GA, globular actin; GOQDs, graphene oxide quantum dots; GOs, graphene oxide nanosheets.

Tryptophan (Trp), as a fluorophore sensitive to the surrounding micro‐environment, was present in the peptide chain of GA.^26^ Thereby, the fluorescence spectrometer will be employed to probe the tertiary structure change of GA caused by the polymerization events (Figure 3). As we know, the polymerization of proteins often leads to spatially tight packing of polypeptide chains, which will in turn trigger the quenching of the fluorophore involved in the side‐chains of amino acid residues. Namely, the polymerization of proteins often intimately correlates with the quenching of fluorophore present in the proteins, and what's more, there might be a relationship between the polymerization level of the protein and the degree of quenching of the fluorophore. As expected, appreciable fluorescence quenching for the pure GA was observed with the polymerization process (Figure 3A). Upon addition of GOQDs or GOs into the GA solution, the characteristic emission maxima of GA red‐shifted from 324 nm for the pure GA to 328 nm for the GA mixed with GOQDs and finally to 337 nm for the GA mixed with GOs (Figure 3A–C), the relatively weak red‐shift caused by the addition of GOQDs could be ascribed to the establishment of a weak and small nano‐bio interface between GOQDs and GA due to the weak π‐π conjugate between them, and the appreciable red‐shift resulted from the addition of GOs could be rationalized from the establishment of a strong and large nano‐bio interface between GOs and GA due to the strong π‐π conjugate between them.^27^, ^28^

Apart from the difference in the red‐shift, the addition of GOQDs and GOs also led to a distinct fluorescence quenching degree of GA. We compared the final fluorescence signal (60 min) of them, and as shown in Figure 3D, the GA + GOs group showed more significant quenching than that observed in the GA + GOQDs group. As we all know, GOs are an excellent quenching agent for aromatic amino acids.^29^ Meanwhile, the two‐dimensional GOs have a planar structure and ultrathin thickness with a high interface, which helps to provide a larger nano‐bio interface to conduct reactions. From a dimensional dependency viewpoint, GOs can provide a much larger protein‐binding interface than GOQDs, if we exclude other effects like inserting into and disassembling filaments.^11^, ^12^ To further interrogate the effect of two types of GBNs on the polymerization behavior of GA, the Stern‐Volmer type plots are delineated in Figure 3E, where F_0_ and F represent the fluorescence intensities of GA polymerization in the absence or presence of GOQDs and GOs, respectively. Notably, the pure GA presented typical time‐dependent fluorescence decay, implying the typical polymerization kinetics of pure GA. The GA + GOQDs group exhibited a weakened feature in time‐dependent fluorescence decay, likely due to the disrupted polymerization of GA with the addition of GOQDs. More importantly, the GA + GOs group instead showed no feature in time‐dependent fluorescence decay, largely since the polymerization of GA had been fully blocked at the beginning, again experimentally highlighted the pivotal role of strong and large interface established between GOs and GA in weakening and even blocking the polymerization of GA.

**FIGURE 4:**
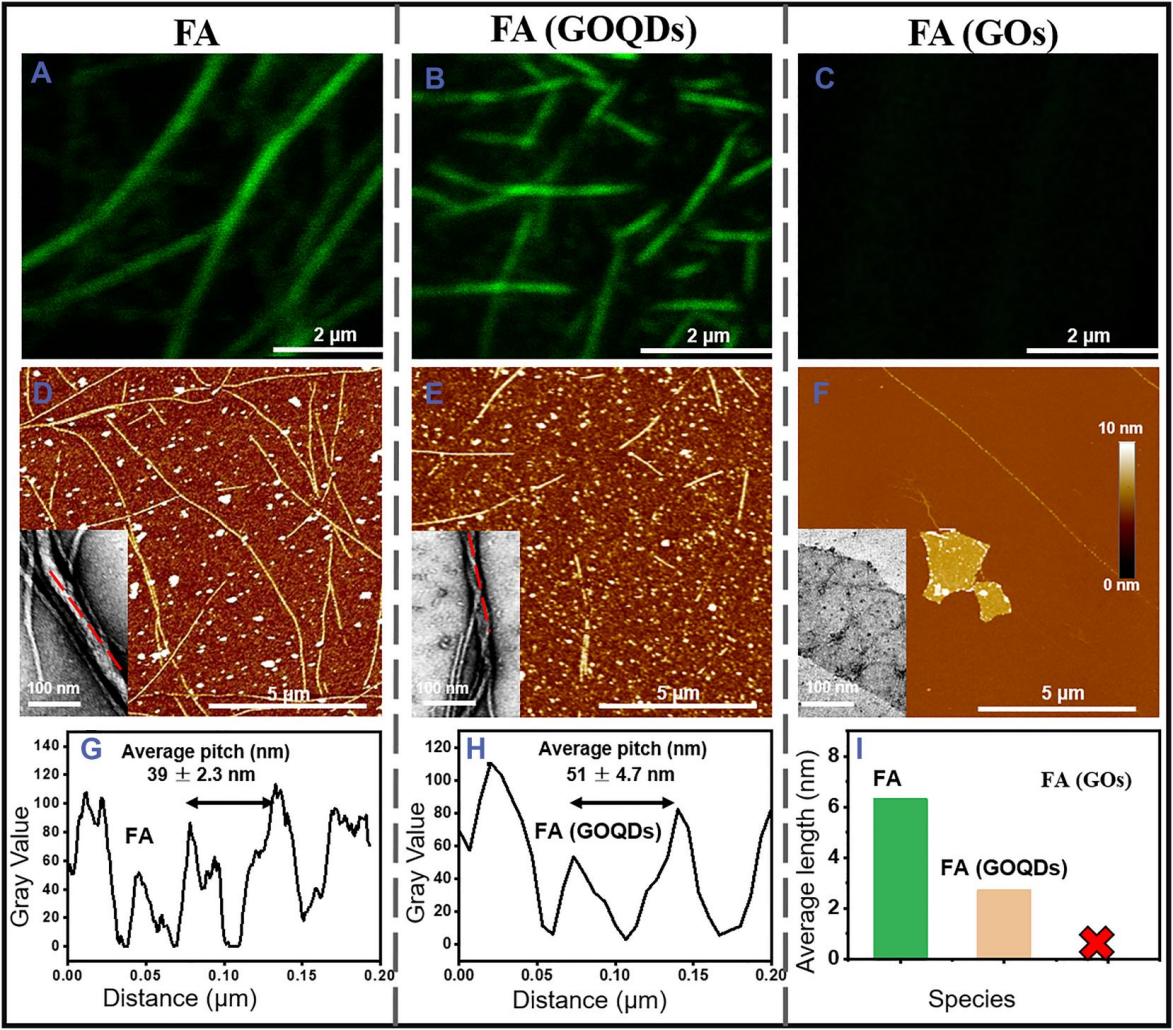
Dimensional effects on actin polymerization. Fluorescence images of FA labeled with Alexa 488‐Phalloidin in the absence (A) and presence of GOQDs (B) and GOs (C). The corresponding AFM images of FA in the absence (D) and presence of GOQDs (E) and GOs (F), and the insets are TEM images for showing the pitches of single filaments. The helix pitch measurements of FA formed in the absence (G) and presence (H) of GOQDs. (I) Statistical analysis of the distribution of FA lengths in the absence and presence of GOQDs or GOs. Data were collected from three independent experiments (*p < 0.05). AFM, atomic force microscope; FA, F‐actin; GOQDs, graphene oxide quantum dots; GOs, graphene oxide nanosheets.

With the aid of a high‐affinity filamentous actin probe, Alexa 488‐Phalloidin,^30^ the polymerization of GA into FA in the presence of GOQDs or GOs was first monitored by the laser scanning confocal microscope (LSCM), and then tracked by AFM and TEM (Figure 4, Figure S3). Here, the native GA served as a control, the LSCM results showed that native GA can polymerize into FA with a few microns in length (Figure 4A,D). However, in the presence of GOQDs, the remodulated GA can only polymerize into short filaments (Q‐FA, Figure 4B,E). Unexpectedly, in the presence of GOs, no actin filament could be detected by LSCM (Figure 4C,F), suggesting that the polymerization of GA toward FA was inhibited due to the introduction of GOs. TEM was leveraged to probe the structural difference between FA and Q‐FA. The FA bundles polymerized by the native GA performed a periodic spiral structure with a helix pitch of about 40 nm (Figure 4D inset, G), which is consistent with the previous report.^31^ However, in the presence of GOQDs, the helix pitch of Q‐FA increased to approximately 50 nm (Figure 4E inset, H). In this study, a larger pitch structure metaphorically represents a reprogramming process of the polymerization of GA, which guides a freakish FA structure (Figure 4I). Both AFM and TEM images showed no appreciable filaments in groups incubated with GOs (Figure 4C). However, some small particles were observed on the surface of GOs (Figure 4F), which may be attributed to the spontaneously adsorbed GA onto GOs. Statistically, native GA polymerized into well‐defined mature FA longer than 5 μm. In the presence of GOQDs, the remodulated GA reprogrammed the polymerization of actin and induced a short construction size in the FA bundles (∼2 μm) (Figure 4I). It appeared that both GOQDs/GOs can redirect the GA in terms of polymerization behavior and filamentous structure, with the GOs displaying a stronger effect on the polymerization propensity of the GA.

The inhibition difference in nano‐bio interface effects on actin polymerization is very clear and confirmed with diverse techniques, consistent with existing geometry/dose‐dependent theories.^16^, ^32^, ^33^ In comparison, GOs can expand a much larger protein‐binding interface than GOQDs, so it is reasonable to predict that GOs show more powerful than GOQDs to perturb the polymerization under the same mass, but similar inhibition effects were also observed if we tuned the exposing areas to the comparable using a 3 times concentration of GOQDs (Figure S4). We proposed a rational theoretical explanation for why GOs have unprecedented inhibition efficiency on GA polymerization, merely the exposing area determining actin polymerization, which does not conflict with the coexisting geometry of dose‐dependent theories. Both GOQDs and actin oligomers are spheres with a high curvature, the nano‐bio interface area (exposing) between these two substances will be greatly reduced compared with that in the case of GOs. Compared with the two‐dimensional GOs, the three‐dimensional GOQDs are smaller in size and have better dispersibility in solution, which makes their interaction with GA much weaker and causes less structural damage. Actin contains tens of aromatic amino acid residues, we proposed that the binding adsorption is due to strong π‐π stacking and hydrophobic interaction between actin and GOQDs/GOs.

### 2.3 Demonstration of GBN with different dimensions on cancer therapies

**FIGURE 5:**
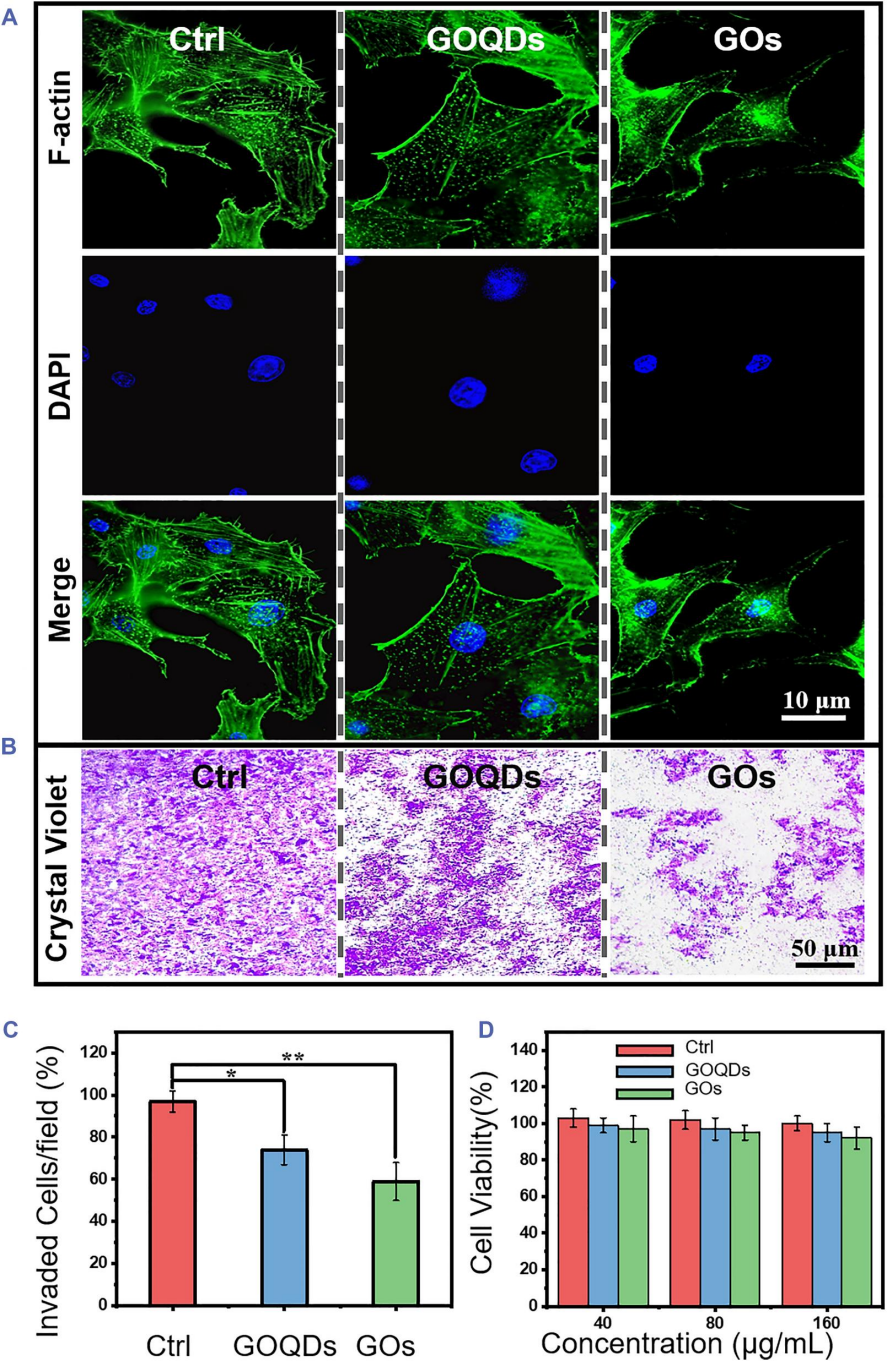
Dimensional effects on cancer therapies. (A) LSCM images of B16F10 cells incubated without or with 80 μg/mL of GOs and GOQDs, respectively. Scale bar = 10 μm. (B) Microscopy images for the transwell assays of B16F10 cell migration. Scale bar = 50 μm. (C) The statistical result of cell migration from (B). (D) Cell viability of B16F10 cells with/without incubating 80 μg/mL GOQDs or GOs for 24 h. Indicating a significant difference between the treatments (*p < 0.05). GOQDs, graphene oxide quantum dots; GOs, graphene oxide nanosheets; LSCM, laser scanning confocal microscope.

As we know, actin filaments can particularly form a network to regulate cell migration. In this sense, the effects of GOQDs and GOs on actin polymerization will finally influence the cytoskeleton formation, which could significantly affect the migration and metastasis of cancer cells. To achieve this goal, we used the murine melanoma cell line, B16F10 cells, as a typical model of cancer cells to investigate whether GBNs will also affect the cell migration. With the help of Alexa‐Fluor 488‐labeled phalloidin, cells can be easily viewed by LSCM. The typical structure of actin filaments was well‐ordered bundles (Figure 4A). When B16F10 cells were pretreated with GOQDs or GOs for 12 h, the actin filaments showed non‐isotropic behavior, and the fluorescence intensity of actin filaments was reduced (Figure 5A), indicating the inhibition effect of GBNs. Tian et al. reveled that GOs can inhibit cell migration by directly disrupting actin filaments. Our results also confirmed that GOs can bind more actin proteins and show more significant structural alterations on the cytoskeleton than GOQDs. Transwell assays were also performed to test the effects of GOQDs and GOs on the migration of B16F10 cells. Compared with the control group, the migration abilities of B16F10 cells which were exposed to identical concentrations of GOQDs or GOs (100 μg/mL) were significantly reduced (Figure 5B,C), suggesting that both GOQDs or GOs can reduce cancer cell migration by reprogramming the actin cytoskeleton. Like the in vitro experiment results, GOs showed a stronger inhibition effect than GOQDs. As per the recent report by Hu's group, GBNs can significantly retard the migration of hematopoietic stem cells (HSCs) by deactivating HSCs, wherein GOs could induce the oxidative stress rather than GOQDs owing to their geometry.^34^ Our results argue that the main reason to inhibit the B16F10 cells migration was that GOQDs/GOs reprogrammed the polymerization of GA, which is a nano‐bio interface dependent regulation. GOs with an excellent nano‐bio interface exhibited an unprecedented inhibition efficiency, led by an effective synergistic effect between the collapsed GA and GOs. The deeply frustrated GA reprogrammed the polymerization of the cytoskeleton and further inhibited the migration of cancer cells. Cells treated for 24 h were still viable and could be efficiently stained by MTT. After being exposed to GOQDs or GOs for 24 h, B16F10 cells showed good viability even at a high concentration (140 μg/mL) (Figure 5D). The GOs induced only minimal toxic responses even at high concentrations, which indicated that GOQDs and GOs did not trigger apoptosis for B16F10 cells.

**FIGURE 6:**
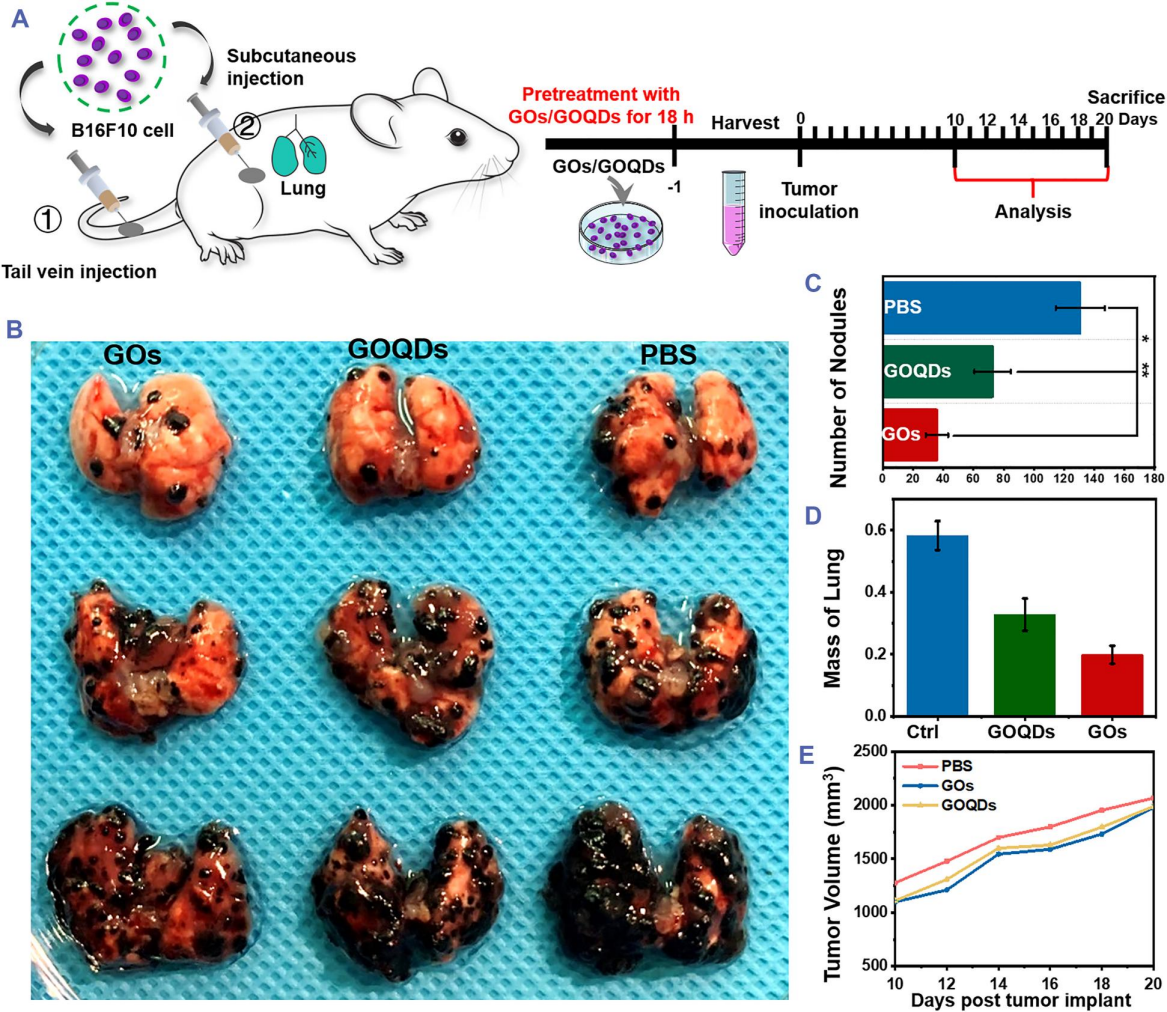
Dimensional effect of GO materials in the metastatic B16F10 tumor model. (A) Diagram illustrating the experimental scheme for the B16F10 metastatic tumor model. B16F10 cells (pre‐treated with PBS, GOs and GOQDs respectively) were injected by tail vein or subcutaneously. Mice were sacrificed on day 20, and lungs (B) were harvested and analyze. The black mass indicated melanoma nodules. (C) Lung nodules larger than 2 mm in diameter were manually counted. (D) Lung weights from different groups. (E) The tumor‐volume curves of the B16F10 tumor‐bearing mice model handled with PBS, GOs or GOQDs, respectively. Bars indicated the mean tumor weight ± SEM (*p < 0.02). GOQDs, graphene oxide quantum dots; GOs, graphene oxide nanosheets.

Melanoma, a fatal form of skin cancer, is a tumor generated from melanocytes. Melanoma is characterized by high invasion and metastatic rates, and its eradication is challenging. Hence, B16F10 melanoma is the gold standard for evaluating melanoma metastasis in clinical trials. Through subcutaneous or tail vein injection, we generated the syngeneic B16F10 model to investigate how GOs and GOQDs hindered actin cytoskeleton polymerization and influenced cancer cell migration (Figure 6A). B16F10 cells were pre‐treated with GOs and GOQDs for approximately 18 h prior to subcutaneous and tail vein injections, respectively. The control groups were administered PBS buffer. Because of the propensity of malignant tumors to metastasize to the lungs, the lungs were harvested 20 days after B16F10 cell injection. Visible nodules (>2 mm in diameter) were examined and manually numbered in all groups as shown in Figure 6B, and the photos clearly demonstrate the significant hypermelanosis of migrating B16F10 cells in the lungs. The GOs and GOQD groups had less lung metastatic nodules than the PBS group, and the GO groups showed the least nodules according to statistical analysis (Figure 6C). The body weight and tumor size were monitored starting on the eighth day and every other day after that. The body weight data suggest that no toxicological reaction occurred in any of the experimental groups (Figure S5A,B). After 20 days, there was a significant difference in lung weight between the control groups and GOQD groups as well as GO groups (Figure 6D), indicating a distinct difference in metastasis burden. The tumor volume growth curves reveal that for 20 days, the mice in all three groups expanded over 200 mm in diameter (Figure S5C, Figure 6E). The tumor weights of these three groups were similar because B16F10 melanoma subcutaneous development is dictated by cell viability (Figure S5D). These data demonstrate that the decrease in metastatic nodules is independent of the activity of the B16F10 cells described in this study. Comprehensive the results of mechanism validation in vitro and vivo proved that the two‐dimensional GOs demonstrated a striking nano‐bio interface, which is an effective inhibitor of cancer cell metastasis caused by actin polymerization inhibition. The superb nano‐bio interface in GO‐based materials can exploit their distinctive properties to profoundly affect actin polymerization and further inhibit the metastasis of B16F10 cells.

The actin cytoskeleton normally acts as a dynamic structural support and a functional executive structure in cellular metabolism as well. Generally, FA with highly ordered structures is thermodynamically stable except when they are exposed to substances that can strongly bind to them.^32^, ^35^ With different substances, cells behave differently in response and FA will rearrange accordingly. In this work, when GA was incubated with GOQDs, only short FA formed, which means that GOQDs can suppress the elongation of FA. In contrast, GOs can inhibit the polymerization of GA seriously, so no classical FA was found at all. Our study highlighted the pivotal role of the dimensional bioeffects of GBNs in modulating the polymerization of GA as well as in inducing different proliferation styles of cancer cells. All the data might provide a promising direction for GBN‐based therapy for tumor metastasis by rational design of the morphology of GBNs.

## 3 CONCLUSIONS

In summary, we demonstrated the pivotal role of dimensional coupling between GBNs and GA in anticancer and anti‐tumor effectiveness. Our results clearly showed the lower dimension possessing more potent effects, which was ascribed to the more unfolded nano‐bio coupling interface. With the strong coupling between cellular proteins and bio‐materials, the protein conformation at both secondary and tertiary structural levels could be significantly disturbed, which further influences their self‐assembly, for example, actin polymerization. By focusing on the actin proteins and their polymerization, we successfully proved the dimensional effect of GOs modeled bio‐materials from killing cancer cells to anti‐tumor metastasis. As expected, lower dimensions can exert more powerful effectiveness in disturbing cytoskeleton formation both for in vitro and in vivo experiments. Based on the interaction of GBNs with actin protein in vitro and in vivo, we believe that the nano‐bio interface of GBNs should be carefully considered in tumor therapy, particularly for the rational design of GBNP‐based drug delivery systems. We think this work provides a different viewpoint, that is, structural dimension, for in‐depth understanding the nano‐bio coupling interface and rational design of novel bio‐materials for tumor therapies.

## 4 EXPERIMENTAL SECTION

Experimental details are provided in the Supporting Information.

## AUTHOR CONTRIBUTIONS

Feng Zhang, Yi Zhang, and Qiqige Du initiated the concept and designed the experiments. Qiqige Du and Jiaqi Lian conducted the experiments. The following individuals contributed to the data analyses, scientific discussions, and authorship of this paper: Jun Guo and Na Li. All authors revised the manuscript and provided helpful comments.

## CONFLICT OF INTEREST STATEMENT

All authors declare that there are no competing interests.

## ETHICS STATEMENT

The animal protocol and experimental procedures were approved by the Animal Research and Ethics Committee of Wenzhou Institute of the University of Chinese Academy of Sciences (Approval Issue No. WIUCAS22041501).

## Supporting information

Supporting Information S1
