# Supplementary material for: Dimensional effect of graphene nanostructures on cytoskeleton‐coupled anti‐tumor metastasis
Source: Smart Med. 2023 Aug 3;2(3):e20230014. doi: 10.1002/SMMD.20230014 (PMC11235939; doi:10.1002/SMMD.20230014)
Supplement: Supplementary file 1 — Supporting Information S1 [file SMMD-2-e20230014-s001.docx]

**Dimensional effect of graphene nanostructures on cytoskeleton-coupled anti-tumor metastasis**

Qiqige Du^1^, Na Li^2^, Jiaqi Lian^2^, Jun Guo^1,^ *, Yi Zhang^3,^ *, Feng Zhang^1, 2,^ *

^1^ Wenzhou Institute, University of Chinese Academy of Sciences, Wenzhou 325001, China.

^2^ Key Laboratory of Optical Technology and Instrument for Medicine, Ministry of Education, University of Shanghai for Science and Technology, Shanghai 200093, China.

^3^ Shanghai Advanced Research Institute, Chinese Academy of Sciences, Shanghai 201203, China

* Corresponding e-mails: [fzhang@usst.edu.cn](mailto:fzhang@usst.edu.cn) (F. Z.); [zhangyi@sinap.ac.cn](mailto:zhangyi@sinap.ac.cn) (Y. Z.); [guojun-nbm@wiucas.ac.cn](mailto:guojun-nbm@wiucas.ac.cn) (J. G.)

**Experiments**

**Materials**

GA powder was purchased from Cytoskeleton Inc. (USA). GA (>99% purity) was directly used without further purification. The muscovite mica was purchased from Meifeng Mica Industry Corp. (Sichuan, China). GOQDs and GOs were commercially available from XF Nano Inc. (Nanjing, China). ATP and guanidine carbonate were purchased from Sigma. Alexa 488-Phalloidin and Hoechst 33342 were purchased from Beyotime (China). Other chemicals were from China Chemicals Ltd Inc, used without any further purification.

**Preparation of stock solutions**

The actin protein powder was dissolved in Tris-HCl buffer (pH 8.0, 5 mM) containing 0.2 mM CaCl_2_, 0.2 mM ATP, 5% (w/v) sucrose, and 1% (w/v) dextran with a concentration of 10 mg/mL. Prepared actin solution was snap-frozen in liquid nitrogen and saved at -70 ℃. Prior to the experiment, the GA solution was diluted to 0.4 mg/mL with general actin buffer (GB), which contains 5 mM Tris-HCl (pH 8.0), 0.2 mM CaCl_2_, 0.2 mM ATP, and 0.5 mM DTT. The GA solution was incubated under ice for one hour, making the possibly existing actin oligomers depolymerized. Then the GA solution was centrifuged at 4 °C (Hitachi, Japan) at 14,000 rpm for about 15 min, and then the supernatant was moved to a new microfuge tube and diluted with GB when used. Actin was polymerized at room temperature with PB (10 × strength), which consisted of Tris-HCl (pH 7.5, 100 mM), 20 mM MgCl_2_, 500 mM KCl, 50 mM guanidine carbonate, and 10 mM ATP.

**DLS**

Both the hydrodynamic diameter and the zeta potential measurements were carried out on a dynamic light scattering (DLS) instrument (Malvern Zetasizer Nano ZS90, Malvern Instruments Ltd., Worcestershire, UK) equipped with a quartz or disposable cuvette (DTS0012, the minimum sample volume is 1 mL) at 25.0 °C over 100 scans. The final concentration of GA solution was adjusted to ~100 μg/mL, and the final concentration of both GOQDs and GOs was 100 μg/mL.

**AFM and TEM**

The mixed solution of GA (~ 40 μg/mL) and GOs (100 μg/mL) was co-incubated with PB for about 1 hour, then 10 μL of the above solution was deposited on mica substrate and dried with nitrogen gas. The GOs (100 μg/mL) were absorbed on the surface of mica in GB, then GA (40 μg/mL) was added, and then imaged. After 50 min, PB was added, and imaged to monitor the polymerization process. The mixture of GA (40 μg/mL) and GOQDs (100 μg/mL/300 μg/mL) was recorded, then PB was added, and the liquid mode was also employed to monitor the polymerization process. AFM was performed on commercial equipment (Multimode 8, Nanoscope V, Veeco) using a large scanning size scanner (J type) and a liquid cell. All the cantilevers were purchased from Olympus Company (BL-AC40TS-C2 Biolever mini) with an average spring constant of 0.09 N/m, and the oscillation frequency was around 25 kHz. All the images were obtained in tapping mode, and all the *in-situ* images were obtained in liquid mode.

Transmission electron microscopy (TEM) samples were prepared by dropping a 10 μL sample solution onto a carbon-film-coated copper grid. GA (100 μg/mL) was premixed with GOQDs (100/300 μg/mL) or GOs (100 μg/mL) for about half an hour, following with the addition of PB to induce the polymerization of GA for about one hour at room temperature, and the control group was only polymerized with PB. The TEM images have been captured on a FEI Tecnai G2 20TWIN (200 kV) TEM.

**Far-UV CD spectroscopy**

The secondary structure of GA co-incubated with GOQDs/GOs was evaluated by far-UV CD spectroscopy. All CD spectra were recorded using a commercial Chirascan-auto qCD (Applied Photophysics, UK). CD spectra of the mixture of GA (100 μg/mL), GOQDs (100 μg/mL) and GOs (100 μg/mL) in general buffer were recorded at 25 ℃ with slits as 0.5nm. The used cuvette diameter was 0.1 cm. The samples were scanned from 197 nm to 260 nm, with the step size of 1nm and bandwidth as 0.5 nm. The change of secondary structures of GA was analyzed by the CDNN program.

**Fluorescence spectroscopy**

All the fluorescence spectroscopy measurements were performed on a commercial fluorescence spectrometer (Edinburgh Instruments FS920, UK). The excited wavelength was 280 nm, and the slits for both excitation and emission were 10 nm. The sample’s concentration was consistent with that in the CD experiment.

**Cytoskeleton staining**

Cells incubated with GOQDs/GOs (40/80/160 μg/mL) were fixed with 4% paraformaldehyde for about 10 min and then incubated with Triton X-100 in PBS for 5 min. 3% bovine serum albumin (BSA) was added as a blocking solution and incubated for 45 min. Then cells were incubated with Alexa 488-Phalloidin in PBS for about 2 hours and washed with PBS for three times. Hoechst 33342 was added to the cells and incubated for 15 min, washed again, and detected by confocal microscope (Ti-E; Nikon, Japan).

**Cell migration assays**

Transwell assays were employed to measure the migration of B16F10 cells. Cells cultured with GOQDs (100 μg/mL) and GOs (100 μg/mL) were collected separately. Then, resuspended cells (200 μL, 5 × 10^4^) were added to culture insert, and medium containing 5% FBS was added to the wall. After 12 hours of culture (37 °C with 5% CO_2_), cells were stained with 0.1% crystal violet, then erase the cells which did not pass through the membrane. The crystal violet was washed out with 33% acetic acid. Cells were measured at 570 nm to detect the number of migrated cells.

**MTT assay**

In this assay, B16F10 cells were seeded in 96-well plates (1 × 10^4^) and incubated for 24 hours (37 °C with 5% CO_2_). A Series of diluted GOQDs (40/80/160 μg/mL) or GOs (40/80/160 μg/mL) were added, respectively. After 18 hours of incubation, MTT was mixed with each well and cultured about 4 hours (final concentration 0.5mg/mL). The absorbance value was measured at 570 nm. All experiments were repeated for three times.

**Experimental lung metastasis model**

The animal protocol and experimental procedures were approved by the Animal Research and Ethics Committee of Wenzhou Institute of the University of Chinese Academy of Sciences. Female C57BL/6 mice (5-6 weeks old) were purchased from Shanghai SLAC Laboratory Animal Co., Ltd. (Shanghai, China). All the experiments mice were randomly assigned into three injection groups of three mice each. B16F10 cells were pretreated with GOs (100 μg/mL) or GOQDs (100 μg/mL), respectively for 18 hours. Tumor inoculation were created by two different approaches: (1) for subcutaneous injection, 100 μL of pretreated B16F10 (5×10^5^) cells in phosphate-buffered saline (PBS) were injected into the right beneath the hip; (2) for tail vein injection, 5×10^5^ B16F10 cells in 100 μL PBS were injected into the tail vein of mice. On the eighth day, and every other day thereafter, mice weight and tumor size (subcutaneous group) were measured. At the same time points, the control mice received identical PBS buffer injections. Melanomas were isolated and weighed, and lungs were surgically removed for the metastasis assay at the end of the experiment.


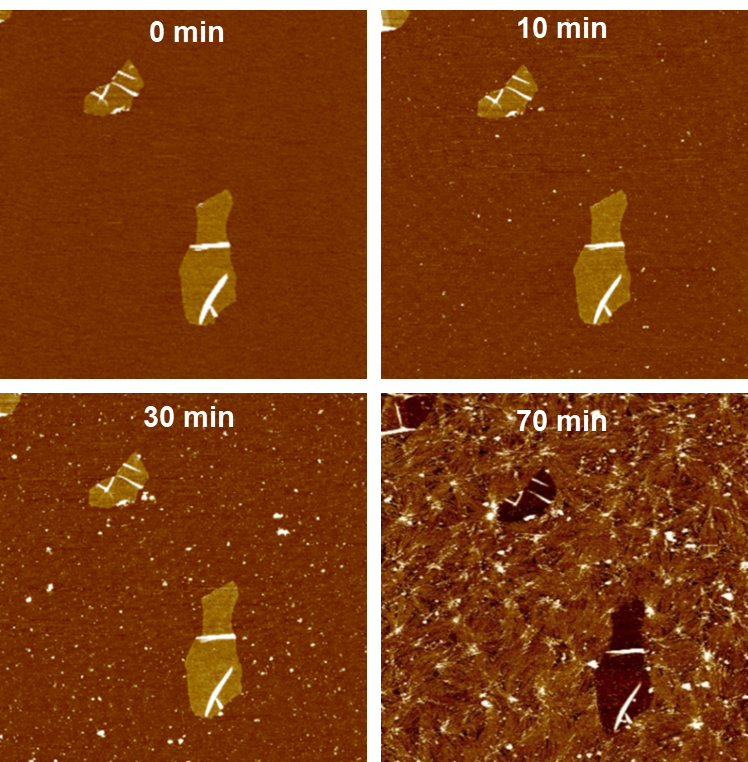


**Figure S1.** ***In-situ* observation of actin polymerization on two-dimensional substrates.**


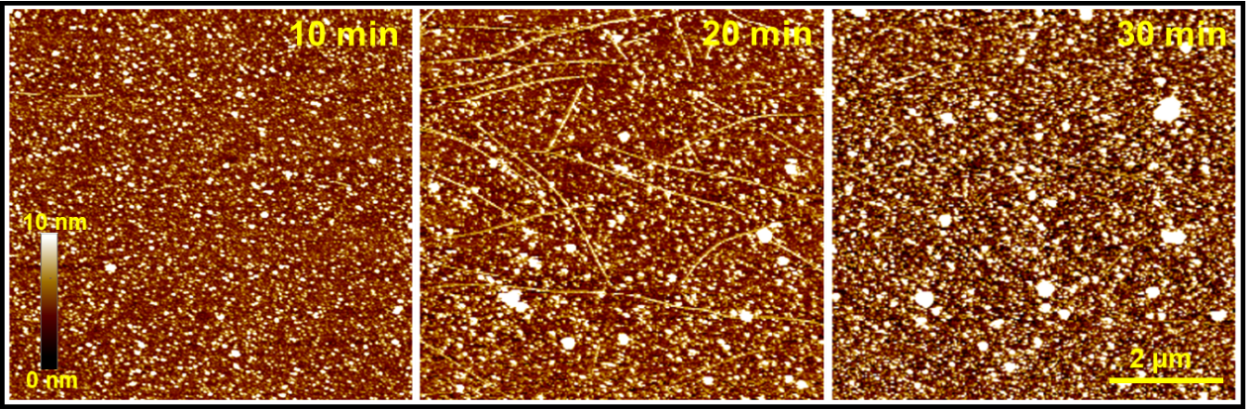


**Figure S2. In situ AFM images of GA assembled with three dimensional GOQDs in liquid phase. With PB, just a few short and scattered filaments were observed, which appears to be considerably weaker than the natural bundle filaments, since they were quickly disassembled during the tip scanning procedure.**


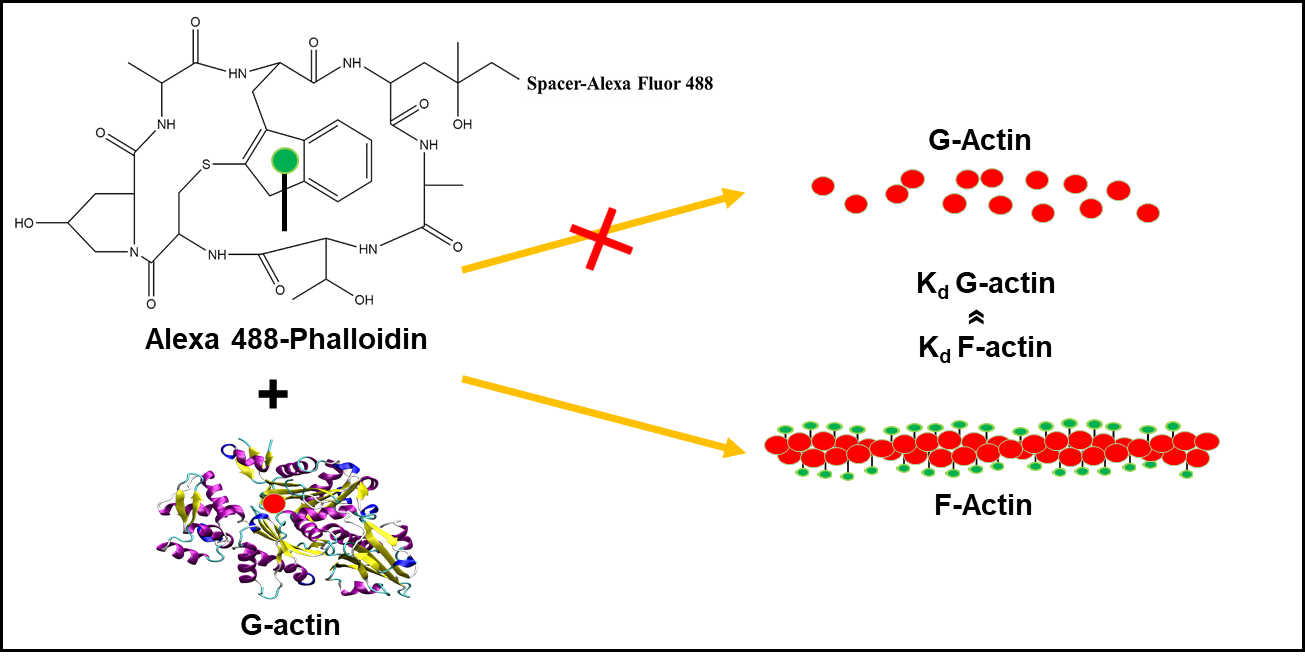
**Figure S3. Mechanistic diagram of Alexa 488-Phalloidin specific labeling F-actin. Alexa 488-Phalloidin is a high-affinity filamentous actin probe with strong affinity that is typically used to mark the FA and stabilize mature FA. Kd represents the dissociation constant.**


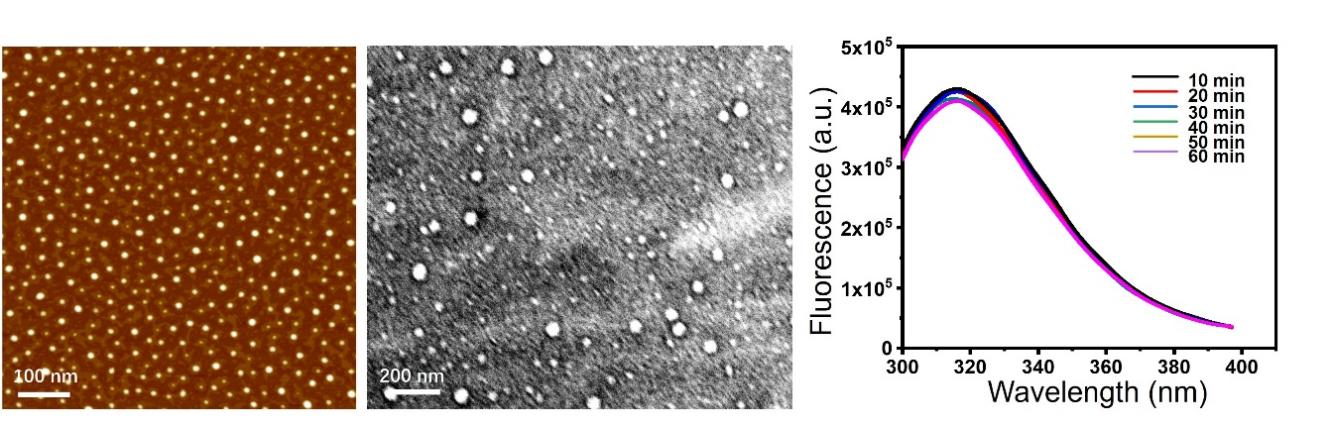


**Figure S4. Exposing bio-nano interface area-depended inhibition effects. AFM (left) and TEM (middle) images and dynamic fluorescence spectroscopy (right) for GA after incubating with GOQDs at a concentration of 150 μg/mL, 3 times that of the other in vitro experiments (50 mg/mL).**


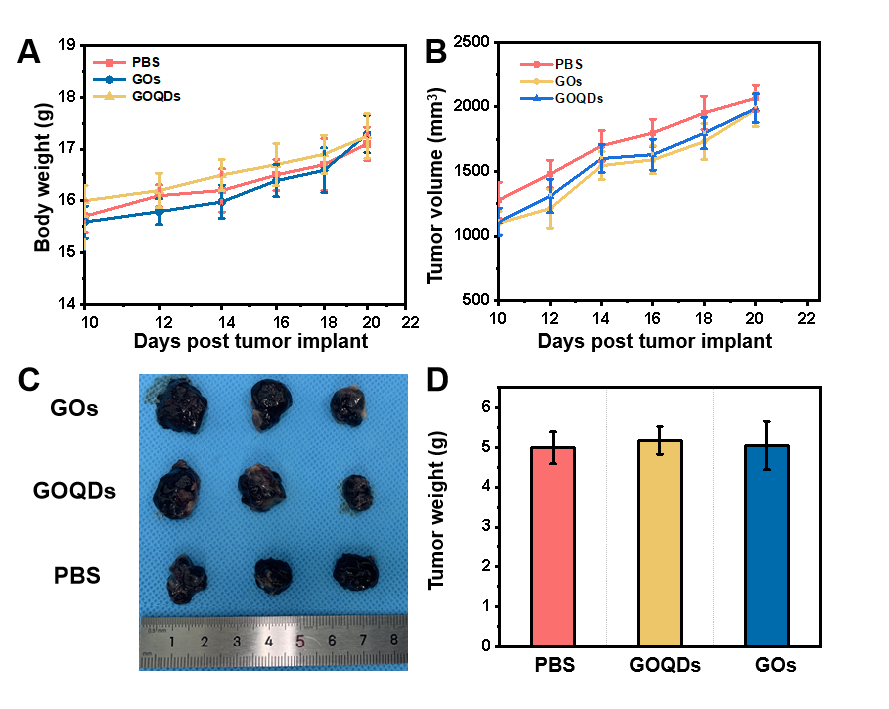


**Figure S5. The suppression effect of GOs and GOQDs in the metastatic B16F10 tumor model. The body weights of the subcutaneous injection group (A) and the Tail vein injection group (B). After mice were sacrificed on day 20, tumors (C) were harvested for imaging analysis. (D) Tumor weight analysis.**
